# A cost-effective adaptive repair strategy to mitigate DDoS-capable IoT botnets

**DOI:** 10.1371/journal.pone.0301888

**Published:** 2024-12-26

**Authors:** Jiamin Hu, Xiaofan Yang

**Affiliations:** School of Big Data & Software Engineering, Chongqing University, Chongqing, China; University of Carthage National School of Engineers of Carthage (ENICarthage) / Higher School of Communications of Tunis (Sup’Com), TUNISIA

## Abstract

Distributed denial of service (DDoS) is a type of cyberattack in which multiple compromised systems flood the bandwidth or resources of a single system, making the flooded system inaccessible to legitimate users. Since large-scale botnets based on the Internet of Things (IoT) have been hotbeds for launching DDoS attacks, it is crucial to defend against DDoS-capable IoT botnets effectively. In consideration of resource constraints and frequent state changes for IoT devices, they should be equipped with repair measures that are cost-effective and adaptive to mitigate the impact of DDoS attacks. From the mitigation perspective, we refer to the collection of repair costs at all times as a repair strategy. This paper is then devoted to studying the problem of developing a cost-effective and adaptive repair strategy (ARS). First, we establish an IoT botware propagation model that fully captures the state evolution of an IoT network under attack and defense interventions. On this basis, we model the ARS problem as a data-driven optimal control problem, aiming to realize both learning and prediction of propagation parameters based on network traffic data observed at multiple discrete time slots and control of IoT botware propagation to a desired infection level. By leveraging optimal control theory, we propose an iterative algorithm to solve the problem, numerically obtaining the learned time-varying parameters and a repair strategy. Finally, the performance of the learned parameters and the resulting strategy are examined through computer experiments.

## 1 Introduction

The Internet of Things (IoT) is a network of interconnected heterogeneous entities that enables data exchange and services without human intervention [1]. As large-scale IoT devices have limited resources and few built-in security principles, an increasing number of IoT devices are now being targeted by attackers [2, 3]. Distributed denial of service (DDoS) attacks launched by botnets are considered one of the prime threats [4–6]. An IoT device can be converted into a controlled IoT bot by an attacker exploiting software or hardware vulnerabilities in the device. Furthermore, the IoT bot can spread botware through port scanning, and then a botnet consisting of these massively infected IoT devices is established. These bots in the IoT botnet send DDoS traffic to one or more target servers at the command of a bot master, aiming to render target servers unusable. Since IoT devices usually remain online and connected to the internet for extended periods, DDoS-capable IoT botnets are more destructive than traditional botnets. Therefore, there is an urgent need to mitigate DDoS attacks by effectively inhibiting the spread of botware.

### 1.1 Motivation

Epidemic modeling-based optimal control theory provides direction on how to effectively inhibit botware propagation. Epidemic modeling stems from the process of describing and predicting the propagation of various infectious diseases in human populations, which is defined as the transition from one state to another as a result of exposure to some phenomenon [7]. The epidemic modeling-based optimal control theory aims to use mathematical models to describe the dynamics of epidemic spread and apply optimization techniques from control theory to determine the most effective intervention measures for achieving specific control objectives [8]. Since the spread process of botware is extremely similar to that of infectious diseases, most of the effective control strategies developed to inhibit botware propagation are based on epidemic modeling [9–14].

However, epidemic modeling-based optimal control involves several parameters (e.g., infection rate) that are crucial for maximizing control effectiveness. Previous efforts to achieve such control effects typically rely on heuristic parameters, i.e., inferred from experience rather than determined through real-world scenarios. In reality, these parameters can dynamically change due to the interaction of various factors, including natural and human elements. Hence, more work is required to accurately predict and control spread dynamics during botware propagation.

In this study, we developed a data-driven optimal control model to obtain an Adaptive Repair Strategy (ARS) using parameters learned from actual network traffic. Specifically, when an IoT network is suspected of harboring DDoS-capable IoT botnets, security systems need to repair the IoT network under a DDoS attack through packet filtering, service or network topology reconfiguration, etc. The repair cost at all times needs to be determined by the infection level of the botware. The ARS aims to predict the botware propagation dynamics based on learned time-varying parameters, thereby automatically adjusting the repair cost (bandwidth, computing, memory resources, or storage). Therefore, the goal of this paper is to address the following problem:

*ARS problem: Develop a cost-effective ARS for IoT networks that can control IoT botware propagation to a desired infection level.*

### 1.2 Contributions

The main contributions of this paper are sketched as follows:

- An IoT botware propagation model is proposed to characterize the evolution of the expected network state of the IoT network. Based on network traffic data, K-Nearest Neighbor (KNN) is used to obtain the reported network state of the IoT network at multiple discrete time slots. An algorithm for obtaining the reported network states, which we refer to as the *RIR algorithm*, is presented. According to the expected and reported network state, detailed modeling processes for parameter learning and prediction of the propagation model are elaborated.
- We model the ARS problem as a data-driven optimal control model, which we refer to as the *ARS model*. An algorithm for solving the ARS model, which we refer to as the *CED algorithm*, is presented. The performance of the proposed algorithms is inspected through numerical experiments, showing that our approach can effectively fit and predict propagation while controlling it within a desired infection level.

The remainder of this paper is structured as follows: Section II reviews the related work. Section III proposes a data-driven optimal control model for the ARS problem. Section IV designs an algorithm for solving the ARS model. Section V examines the performance of the proposed algorithms. This work is closed under Section VI.

## 2 Related work

In this section, we will review related work on modeling malware propagation with epidemic models. Next, we provide an overview of various works on optimal control theory based on malware propagation models.

Malware propagation models are used to characterize the evolution of the expected state of networks over time. Xia et al. [15] developed a state evolution model for social IoT networks to study the impact of node identification ability and node spread capability on botware propagation. Their model was established based on the spread, exposed, ignorant, and recovered model. del Rey et al. [16] proposed a state evolution model for complex networks to simulate malware propagation. They reformulated the compartmental and deterministic global susceptible-infectious-recovered model. Chen et al. [17] presented a hypergraph-based state evolution model for large-scale wireless networks to describe malware propagation. Their model was established based on the susceptible, infected, and recovered model, which obtained the malware outbreak threshold. Carnier et al. [18] established a state evolution model for IoT networks to derive the exact Markov chain for the random propagation of malware. Their model was used based on the susceptible, infected, and susceptible model. Awasthi et al. [19] analyzed the state evolution process in wireless sensor networks to investigate the multi-malware propagation dynamics. They considered the susceptible-exposed state 1, exposed state 2-infectious-recovered model. Dou et al. [20] captured the spatial-temporal propagation behavior and local interaction of botware propagation in mobile wearable IoT networks, which was based on one susceptible state and two different infected states.

Optimal control theory is an applied mathematics discipline dedicated to finding a strategy that governs the evolution of a dynamical system so that a given performance index is optimized [21]. This theory has widespread applications in inhibiting botware propagation when combined with epidemic models [12]. Based on the susceptible, un-informed, and informed network state, Farooq et al. [10] investigated the device-to-device (D2D) propagation of botware in wireless IoT networks. Based on the susceptible-infected-recovered malware propagation model, Kumari et al. [11] presented an optimal policy to control the malware spreading on mobile wireless sensor networks effectively. They determined the equilibrium state of botware infection and information propagation to obtain the optimal patching rates. Based on the botware propagation (susceptible-infected-susceptible) model, Wang et al. [13] addressed the critical threshold of the onset of botware propagation and the lowest-cost defense strategy in heterogeneous IoT networks. Based on the botware propagation model (with susceptible, latent, and propagated-recovered states), Zhu et al. [14] devised an optimal dynamic recovery rate that combines the cost and the overall infection rate of the IoT heterogeneous devices. Based on the susceptible-infected-susceptible botware propagation model, Chen et al. [9] proposed an optimal defense framework that improves the resilience of the cyber-physical grid by considering strategic attack behavior.

Differential game theory, as an extension of optimal control theory, studies strategic time-varying interactions between informed and reasonable players [22]. Differential game theory, when combined with epidemic modeling, provides a powerful tool for the design of control policies for botware propagation processes in the presence of strategic adversaries. Based on the malware propagation model with four states (susceptible, infectious, dead, and non-cooperative) and using a zero-sum differential game, Zhang et al. [23] deduced optimal dynamic defender and attacker strategies in D2D offloading networks. Based on a malware propagation model with five states (sleeping, attacking, defending, paralyzed attack, and paralyzed defense) and through a zero-sum differential game, Wu et al. [24] gave an optimal network resource competition and strategic interaction between an attacker and a defender in an IoT system. Based on the susceptible-infectious malware propagation model and a malware propagation-aware game, Bi et al. [25] studied high-quality attack and defense strategy pairs for microgrids. Based on a malware propagation model with three states (high-secure, low-secure, and insecure) and a differential game, Gan et al. [26] proposed a cost-effective defense strategy for advanced persistent threats based on equipment classification in industrial IoT.

In the above works, they did not show how the parameters of the malware propagation model are obtained in the real world. Therefore, the technique mentioned above may not be very suitable for the ARS problem. In this paper, on the basis of actual network traffic, we apply the optimal control theory based on a data-driven method to learn the parameters of the botware propagation model to solve the ARS problem. To our knowledge, this work is the first attempt to study botware propagation in IoT networks through a data-driven optimal control theoretic approach. Table 1 compares our survey with the other ones based on malware propagation-based optimal control theory.

**Table 1:** Comparison of different surveys on epidemic modeling-based optimal control theory.

Research Work	Year	Controller	Expected Network State	Control Object	Parameter Learning
Farooq et al. [10]	2019	single	susceptible, un-informed, informed	patching rate	no
Kumari et al. [11]	2021	single	susceptible, infected, recovered	the rate of physical damage or energy exhaustion	no
Wang et al. [13]	2023	single	susceptible, infected, susceptible	patching cost	no
Zhu et al. [14]	2023	single	susceptible, latent, propagated, recovered	recovery rate	no
Chen et al. [9]	2023	single	susceptible, infected, susceptible	cyber defense strategy	no
Zhang et al. [23]	2019	multiple	susceptible, infectious, dead, non-cooperative	user equipments participation rate and patching cost	no
Wu et al. [24]	2022	multiple	sleeping, attacking, defending, paralyzed attack, paralyzed defense	defense competition strength	no
Bi et al. [25]	2022	multiple	susceptible, infectious	repair rate	no
Gan et al. [26]	2024	multiple	high-secure, low-secure, insecure	patching cost	no
Our Survey	2024	single	susceptible, infected, recovered	repair cost	yes

## 3 Modeling the ARS problem

The ARS problem was presented in the first section. This section is dedicated to modeling the ARS problem. First, a botware propagation model is formalized. Second, based on the evolution of the expected network state and the reported network state, the ARS problem is modeled as a data-driven optimal control problem.

### 3.1 Formalizing an IoT network evolution model

Suppose the security system intends to mitigate the negative impact of DDoS-capable IoT botnets. First, the evolution of the network state is expected to be obtained to make predictions regarding potential IoT bots. Then, suspicious IoT bots will be repaired through packet filtering, service, or network topology reconfiguration. Suppose the whole mitigation process starts at the initial time *t* = 0 and terminates at the time *t* = *T*. We refer to *T* as the *adaptive repair period*. For *t* ∈ [0, *T*], let *c*(*t*) denote the repair cost spent involving the suspicious IoT bots at time *t*. We refer to the function *c*, *t* ∈ [0, *T*], as an *adaptive repair strategy* (ARS, for short).

Indeed, it is necessary to establish a mathematical model characterizing the evolution of the expected state of the IoT network over time. To this end, suppose there are three states in an IoT network: (i) *Susceptible*: Nodes that have been detected without botware infection; (ii) *Infected*: Nodes that have been detected with probable botware infection, and they can scan adjacent susceptible nodes to propagate botware and send DDoS traffic; (iii) *Recovered*: Nodes that have been repaired after being infected with botware.

Let *S*(*t*), *I*(*t*), and *R*(*t*) denote the proportion of IoT devices in the *Susceptible, Infected,* and *Recovered* states at time *t*, respectively. We refer to the ordered pair (*S*(*t*), *I*(*t*), *R*(*t*)) as the *expected state* of the IoT network at time *t*.

Below, we establish an epidemic model to characterize the evolutionary process of the expected state over time. For this purpose, we first introduce a set of assumptions and parameters as follows:

1. Due to the propagation of botware, the proportion of susceptible nodes becomes infected at time *t* at the rate of *β*(*t*)*I*(*t*)*S*(*t*), where we call the parameter *β*(*t*) the *botware propagation rate*.
2. Due to repair manipulations, the proportion of infected nodes becomes repaired at time *t* at the rate of $\frac{1}{\gamma }c\left(t\right)$, where (i) *γ* is assumed to be positive and constant, referred to as the *DDoS stealthiness level*; (ii) *c*(*t*) is the *adaptive repair strategy* adopted at time *t* and assumed to be controllable. This hypothesis is justified by the fact that the repair rate increases with *c*(*t*) and decreases with *γ*.

Let $\bar{\beta }$ and $\bar{c}$ denote the common upper bounds on *β*(*t*) and *c*(*t*), respectively. Then this requirement can be formulated as $\beta \left(t\right)\in \left[0,\bar{\beta }\right]$ and $c\left(t\right)\in \left[0,\bar{c}\right]$. For simplicity, let *θ*(*t*) = (*β*(*t*), *c*(*t*)) denote the model parameter set. The admissible set for model parameters *θ* is

$$\begin{array}{c} \Theta =\{\beta \in PC\left[0,T\right],c\in PC\left[0,T\right]:\beta \left(t\right)\in \left[0,\bar{\beta }\right],c\left(t\right)\in \left[0,\bar{c}\right],t\in \left[0,T\right]\}, \end{array}$$ (1)

where *PC*[0, *T*] stands for the set of all the piecewise continuous functions defined on the interval [0, *T*].

These assumptions and parameters imply the following result:

**Theorem 1**. *Under the influence of the ARS c*(*t*), 0 ≤ *t* ≤ *T, the IoT network evolves, obeying the following rule*:

$$\begin{array}{c} \left\{ \begin{array}{cc} \frac{dS(t)}{dt} & =-\beta (t)I(t)S(t),\\ \frac{dI(t)}{dt} & =\beta \left(t\right)I\left(t\right)S\left(t\right)-\frac{1}{\gamma }c\left(t\right)I\left(t\right),\\ \frac{dR(t)}{dt} & =\frac{1}{\gamma }c\left(t\right)I\left(t\right),\\ S(0) & =S_{0},I\left(0\right)=I_{0},R\left(0\right)=R_{0}. \end{array}\right. \end{array}$$ (2)

**Fig 1:**
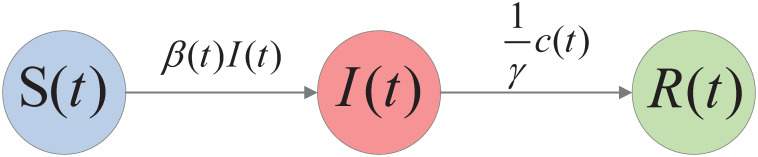
Diagram of the IoT network evolutionary model.

We call Eq (2) an *IoT network evolution model*. See Fig 1 for the diagram of the model.

### 3.2 Getting the reported network state

The key to modeling the ARS problem lies in learning and predicting the parameters of the IoT network evolution model. For this purpose, suppose a reported network state is available based on network traffic data at intermediate observation times for an extended period of time [0, *T*]. The reported network state is derived from the probability of reported infected nodes.

**Remark 1**. *It is possible to acquire the proportion of reported infected nodes at intermediate observation times, as IoT traffic typically exhibits regular and periodic communication patterns, such as packet size, inter-packet interval, and bandwidth. IoT bots can be detected by analyzing these characteristics of historical IoT traffic over an extended period of time*.

There have been attempts to build botnet detectors using machine learning approaches. This paper employs the K-Nearest Neighbors (KNN) classification algorithm to detect IoT bots and obtain a reported network state. The detection process consists of the following phases:

1. *Traffic Capture.* The process involves collecting traffic from IoT devices and recording various features of this traffic, such as the source IP address, source port, destination IP address, destination port, communication protocol, timestamp, etc. All the traffic is then sorted chronologically and used as a training dataset. The training dataset is utilized for binary classification: benign traffic (labeled as “0”) and malicious traffic (labeled as “1”).
2. *Data Pre-processing.* After removing redundant features from the training dataset, all data is converted to numerical values. Let **X**_*n*×*m*_ denote numerical data, where *n* and *m* represent the number of packets from a training dataset and the number of features, respectively. Then, **X**_*n*×*m*_ is normalized so that the values of these features conform to a standard normal distribution using *Z*-score normalization, given by:
  $$\begin{array}{c} {\tilde{x}}_{ij}=\frac{x_{ij}-\mu_{x_{j}}}{\delta_{x_{j}}},\;i=1,\cdots ,n,j=1,\cdots ,m, \end{array}$$ (3)
  where ${\tilde{x}}_{ij}$ is the normalized value of *x*_*ij*_ (*x*_*ij*_ ∈ **X**_*n*×*m*_) in the feature vector **x**_*j*_ of the training dataset, and $\mu_{x_{j}}$ and $\delta_{x_{j}}$ are the mean and standard deviation of the feature vector **x**_*j*_, respectively.
3. *KNN Classification.* The newly arrived traffic is used as the unlabeled testing dataset **Y**_*w*×*m*_, and similarly, *y*_*kj*_ in **Y**_*w*×*m*_ is converted to ${\tilde{y}}_{kj}$ after *data pre-processing*. The Euclidean distance between ${\tilde{y}}_{k}$ (*k* = 1, ⋯, *w*) in **Y**_*w*×*m*_ and ${\tilde{x}}_{i}$ (*i* = 1, ⋯, *n*) in **X**_*n*×*m*_ is calculated as follows:
  $$\begin{array}{c} dist\left({\tilde{x}}_{i},{\tilde{y}}_{k}\right)=\sqrt{\sum_{j=1}^{m}{\left({\tilde{x}}_{ij}-{\tilde{y}}_{kj}\right)}^{2}}, \end{array}$$ (4)
  In the classification phase, *K* is a user-defined constant, and an unlabeled vector ${\tilde{y}}_{k}$ is classified by assigning the label that is most frequent among the *K* packets in ${\tilde{x}}_{i}$ nearest to that query point. In practice, *K* is relatively small. In this regard, cross-validation is an intelligent way to determine the optimal *K* value. It estimates the validation error rate by holding out a subset of the training set from the model-building process.

**Fig 2:**
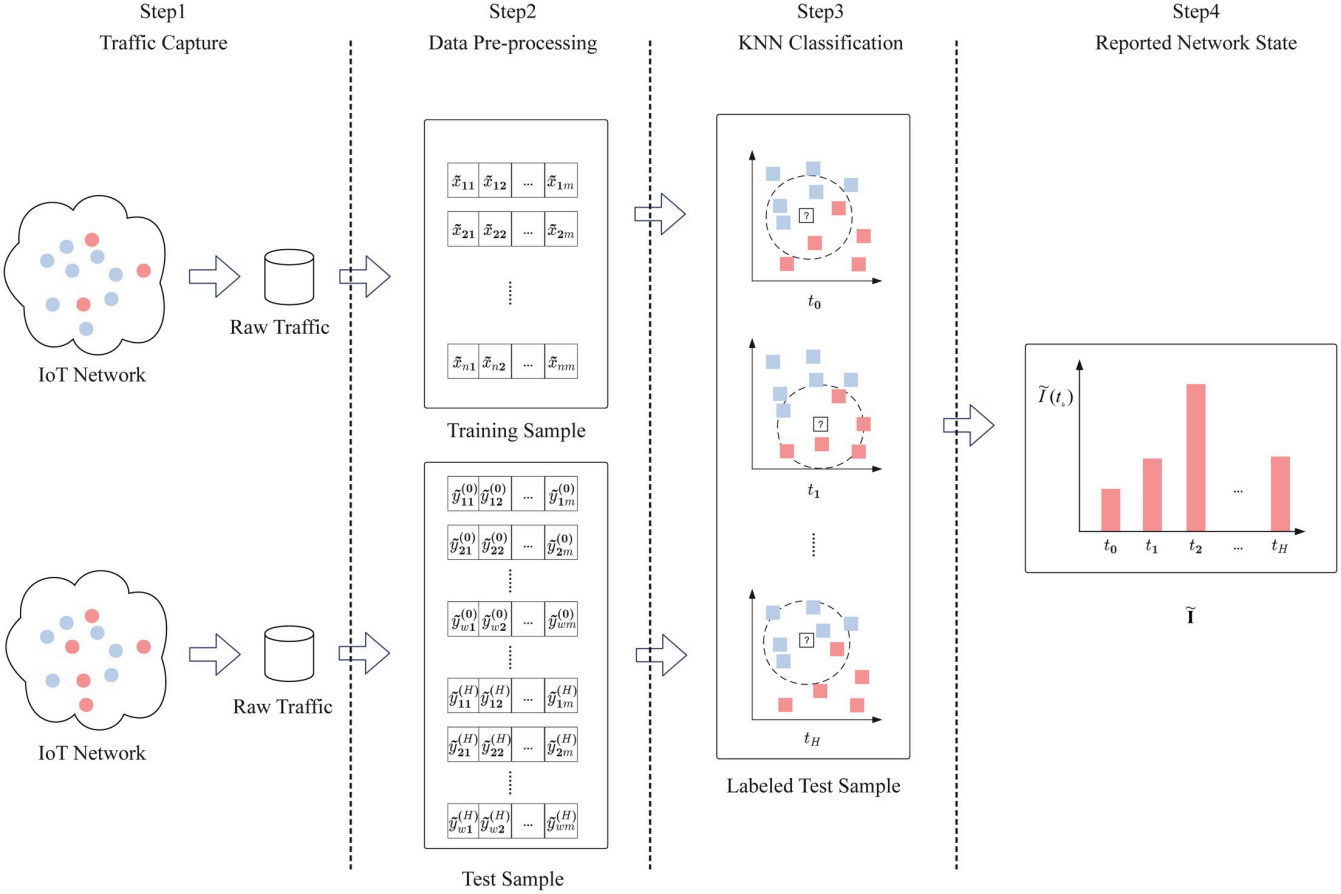
Flowchart of the RIR algorithm.

Let *t*_*h*_ (*h* = (0, ⋯, *H*)) denote the time points for obtaining the reported network state. Then, 0 = *t*_0_ < *t*_1_ < ⋯ < *t*_*H*_ = *T*. Let $\tilde{I}\left(t_{h}\right)$ denote the proportion of IoT devices being infected based on the network traffic data. We refer to the vector

$$\begin{array}{c} \tilde{I}=\left(\tilde{I}\left(t_{0}\right),\tilde{I}\left(t_{1}\right),\cdots ,\tilde{I}\left(t_{H}\right)\right), \end{array}$$ (5)

as the *reported state* of the IoT network at time *t*_*h*_. Based on this, we present the RIR (reported infection ratio) algorithm for obtaining the reported infection state. Refer to Algorithm 1 and Fig 2 for a flowchart of the RIR algorithm.

**Algorithm 1 RIR**

**Input:** A training dataset after data pre-processing, denoted **X**_*n*×*m*_. The time point ***τ*** = (*t*_0_, *t*_1_, ⋯, *t*_*H*_). Testing dataset after data pre-processing until time *t*_*h*_, denoted $Y_{w^{\left(h\right)}\times m}$. The label of all packets in **X**_*n*×*m*_, denoted *l*(**x**_*i*_). A user-defined constant, denoted *K*^(*h*)^. The number of IoT devices, denoted *N*.

**Output:** The proportion of reported state $\tilde{I}$.

1: Calculate the mean $\mu_{x_{j}}$ of **x**_*j*_;

2: Calculate the standard deviation $\delta_{x_{j}}$ of **x**_*j*_;

3: **for** *i* ← 1 to *n* **do**

4:  **for** *j* ← 1 to *m* **do**

5:   ${\tilde{x}}_{ij}=\left(x_{ij}-\mu_{x_{j}}\right)/\delta_{x_{j}}$;

6: *h* ← 0;

7: **repeat**

8:  Calculate the mean $\mu_{y_{j}}^{\left(h\right)}$ of $y_{j}^{\left(h\right)}$;

9:  Calculate the standard deviation $\delta_{y_{j}}^{\left(h\right)}$ of $y_{j}^{\left(h\right)}$;

10:  **for** *k* ← 1 to *w*^(*h*)^ **do**

11:   **for** *j* ← 1 to *m* **do**

12:    ${\tilde{y}}_{kj}^{\left(h\right)}=\left(y_{kj}^{\left(h\right)}-\mu_{y_{j}}^{\left(h\right)}\right)/\delta_{y_{j}}^{\left(h\right)}$;

13:  **for** *k* ← 1 to *w*^(*h*)^ **do**

14:   **for** *i* ← 1 to *n* **do**

15:    $dist\left({\tilde{x}}_{i},{\tilde{y}}_{k}^{\left(h\right)}\right)=\sqrt{\sum_{j=1}^{m}{\left({\tilde{x}}_{ij}-{\tilde{y}}_{kj}^{\left(h\right)}\right)}^{2}}$;

16:   A new sequence $s_{i}^{\left(h\right)}$ of $x_{i}^{\left(h\right)}$ is obtained after sorting $dist({\tilde{x}}_{i},{\tilde{y}}_{k}^{\left(h\right)})$ from smallest to largest;

17:   **for** *ϵ* ← 1 to *K*^(*h*)^ **do**

18:    **if then** $|{⋃}_{l(s_{\epsilon }^{\left(h\right)})=0}s^{\left(h\right)}\left|\leq \right|{⋃}_{l(s_{\epsilon }^{\left(h\right)})=1}s^{\left(h\right)}|$;

19:     $l(y_{k}^{\left(h\right)})\leftarrow 1$;

20:    **else**

21:     $l(y_{k}^{\left(h\right)})\leftarrow 0$;

22:   In ${⋃}_{l(y_{k}^{\left(h\right)})=1}y^{\left(h\right)}$, the number of infected nodes is determined as *N*_*malicous*_;

23:  $\tilde{I}\left(t_{h}\right)=N_{malicous}/N$;

24:  *h* + +;

25: **until** *h* − 1 = *H*;

26: return $\tilde{I}$.

### 3.3 Modeling the ARS problem

Based on the above discussions, we model the ARS problem as a data-driven optimal control problem.

First, based on the reported state $\tilde{I}$, we aim to

- Find the optimal parameter *θ* for a period of time *t* ∈ [0, *t*_*H*−1_] to ensure that the expected network state fit the reported network state, that is, let the proportion of predicted expected state *I*(*t*_*h*_) fit the proportion of reported state $\tilde{I}$ as close as possible.
- Find the desired parameter *θ* for *t* ∈ (*t*_*H*−1_, *t*_*H*_] so as to control IoT botware propagation at time *t*_*H*_ to achieve a desired infection level *ι*.

Then, we define a loss function as follows:

$$\begin{array}{c} P=\sum_{h=1}^{H-1}L(I\left(t_{h}\right))+G\left(I\left(t_{H}\right)\right), \end{array}$$ (6)

where

$$\begin{array}{c} \left\{ \begin{array}{cc} L(I\left(t_{h}\right)) & ={\left[I\left(t_{h}\right)-\tilde{I}\left(t_{h}\right)\right]}^{2},\\ G(I\left(t_{H}\right)) & ={\left[I\left(t_{H}\right)-\iota \right]}^{2}. \end{array}\right. \end{array}$$ (7)

Here the loss function comprises two components: the function *L*, which quantifies the accumulated Mean Squared Error (MSE) between the expected state and the reported state at intermediate observation times, and the function *G*, which gauges the error between the expected state and the desired state at the final time. Therefore, we reduce the ARS problem to the following data-driven optimal control problem:

$$\begin{array}{cc} & \underset{(\beta ,c)\in \theta }{\text{min}}\;P=\sum_{h=1}^{H-1}L(I\left(t_{h}\right))+G\left(I\left(t_{H}\right)\right),\\ & s.t.\{\begin{array}{cc} \frac{dS(t)}{dt} & =-\beta (t)I(t)S(t),\\ \frac{dI(t)}{dt} & =\beta \left(t\right)I\left(t\right)S\left(t\right)-\frac{1}{\gamma }c\left(t\right)I\left(t\right),\\ \frac{dR(t)}{dt} & =\frac{1}{\gamma }c\left(t\right)I\left(t\right),\\ S(0) & =S_{0},I\left(0\right)=I_{0},R\left(0\right)=R_{0}. \end{array} \end{array}$$ (8)

In Eq (8), our objective is to determine the optimal parameters (*β*, *c*) that minimize the loss function *P*, subject to the constraints imposed by the IoT network evolution model described in Subsection 3.1. We denote the data-driven optimal control problem (8) as the ARS model, which is defined by the following 9-tuple:

$$\begin{array}{c} M=(S_{0},I_{0},R_{0},T,\tilde{I},\bar{\beta },\bar{c},\gamma ,\iota ). \end{array}$$ (9)

## 4 Solving the ARS model

In the previous section, we established the ARS model as a mathematical model of the ARS problem. This section is committed to designing an algorithm for solving the ARS model.

### 4.1 An algorithm for solving the ARS model

First, the Hamiltonian function for the ARS model is written as

$$\begin{array}{c} \begin{array}{cc} & H(S,I,R,\theta ,\lambda_{S},\lambda_{I},\lambda_{R})\\ & =\lambda_{S}\left[-\beta IS\right]+\lambda_{I}\left[\beta IS-\frac{1}{\gamma }cI\right]+\lambda_{R}\left[\frac{1}{\gamma }cI\right], \end{array} \end{array}$$ (10)

where (λ_*S*_, λ_*I*_, λ_*R*_) stand for the adjoint variables.

Second, we have the following result:

**Theorem 2**. *Let* *θ be an optimal solution to the problem (8). Let* (*S*, *I*, *R*) *be the solution to the associated IoT network evolution model (2). Then, there exist functions* (λ_*S*_, λ_*I*_, λ_*R*_) *such that the system (11) holds*.

$$\begin{array}{c} \left\{ \begin{array}{cc} \frac{d\lambda_{S}\left(t\right)}{dt} & =\beta \left(t\right)I\left(t\right)\lambda_{S}\left(t\right)-\beta \left(t\right)I\left(t\right)\lambda_{I}\left(t\right),\\ \frac{d\lambda_{I}\left(t\right)}{dt} & =\beta \left(t\right)S\left(t\right)\left[\lambda_{S}\left(t\right)-\lambda_{I}\left(t\right)\right]+\frac{1}{\gamma }c\left(t\right)\left[\lambda_{I}\left(t\right)-\lambda_{R}\left(t\right)\right],\\ \frac{d\lambda_{R}\left(t\right)}{dt} & =0,\\ & t_{h-1}\leq t<t_{h},h=H,\cdots ,1,\\ \lambda_{S}\left(t_{h}^{-}\right) & =\lambda_{S}\left(t_{h}^{+}\right),\\ \lambda_{I}\left(t_{h}^{-}\right) & =\lambda_{i}\left(t_{h}^{+}\right)+2\left[I\left(t_{h}\right)-\tilde{I}\left(t_{h}\right)\right],\\ \lambda_{R}\left(t_{h}^{-}\right) & =\lambda_{R}\left(t_{h}^{+}\right),\\ & h=H-1,\cdots ,1,\\ \lambda_{S}\left(t_{H}\right) & =0,\\ \lambda_{I}\left(t_{H}\right) & =2[I\left(t_{H}\right)-\iota ],\\ \lambda_{R}\left(t_{H}\right) & =0,\\ S(0) & =S_{0},I\left(0\right)=I_{0},R\left(0\right)=R_{0}. \end{array}\right. \end{array}$$ (11)

*Moreover, we have*

$$\begin{array}{c} \theta \left(t\right)\in \text{arg}\;\underset{\tilde{\theta }\in \Theta }{\text{min}}\;H\left(S,I,R,\tilde{\theta },\lambda_{S},\lambda_{I},\lambda_{R}\right),0\leq t\leq T. \end{array}$$ (12)

*for all time* 0 ≤ *t* ≤ *T except* ${\left\{t_{h}\right\}}_{h=1}^{H-1}$.

*Proof*. There exist vector-valued functions (λ_*S*_, λ_*I*_, λ_*R*_) such that system (13) holds. The differential equations in the system (11) are derived by calculating the partial derivatives involved in system (13).

$$\begin{array}{c} \left\{ \begin{array}{cc} \frac{d\lambda_{S}\left(t\right)}{dt} & =-H_{S}\left(S\left(t\right),I\left(t\right),R\left(t\right),\theta \left(t\right),\lambda_{S}\left(t\right),\lambda_{I}\left(t\right),\lambda_{R}\left(t\right)\right),\\ \frac{d\lambda_{I}\left(t\right)}{dt} & =-H_{I}\left(S\left(t\right),I\left(t\right),R\left(t\right),\theta \left(t\right),\lambda_{S}\left(t\right),\lambda_{I}\left(t\right),\lambda_{R}\left(t\right)\right),\\ \frac{d\lambda_{R}\left(t\right)}{dt} & =-H_{R}\left(S\left(t\right),I\left(t\right),R\left(t\right),\theta \left(t\right),\lambda_{S}\left(t\right),\lambda_{I}\left(t\right),\lambda_{R}\left(t\right)\right),\\ & t_{h-1}\leq t<t_{h},h=H,\cdots ,1\\ \lambda_{S}\left(t_{h}^{-}\right) & =0,\\ \lambda_{I}\left(t_{h}^{-}\right) & =\lambda_{I}\left(t_{h}^{+}\right)+\frac{\partial L(I\left(t_{h}\right))}{\partial I},\\ \lambda_{R}\left(t_{h}^{-}\right) & =0,\\ & h=H-1,\cdots ,1,\\ \lambda_{S}\left(t_{H}\right) & =0,\\ \lambda_{I}\left(t_{H}\right) & =\frac{\partial G(I\left(t_{H}\right))}{\partial I},\\ \lambda_{R}\left(t_{H}\right) & =0,\\ S(0) & =S_{0},I\left(0\right)=I_{0},R\left(0\right)=R_{0}. \end{array}\right. \end{array}$$ (13)

*θ* can be obtained according to Eq (12) after plugging in *I* and (λ_*S*_, λ_*I*_, λ_*R*_), which are obtained through Eqs (2) and (11). We solve it by the proximal point algorithm (PPA) [27], that is, for any time *t* ∈ (*t*_*h*−1_, *t*_*h*_), *h* = 1, ⋯, *H*,

$$\begin{array}{c} \begin{array}{cc} & \theta^{\left(l+1\right)}\left(t\right)\\ & =\text{arg}\;\underset{\theta (t)\in \Theta }{\text{min}}\;\{H\left(S^{\left(l\right)}\left(t\right),I^{\left(l\right)}\left(t\right),R^{\left(l\right)}\left(t\right),\theta \left(t\right),\lambda_{S}^{\left(l\right)}\left(t\right),\lambda_{I}^{\left(l\right)}\left(t\right),\lambda_{R}^{\left(l\right)}\left(t\right)\right)\\ & +\frac{1}{2\kappa }\|\theta \left(t\right)-\theta^{\left(l\right)}\left(t\right){\|}^{2}\}, \end{array} \end{array}$$ (14)

where *l* is the index of iteration and *κ* is the step size.

Since *H* is smooth, the above formula is equivalent to

$$\begin{array}{c} \begin{array}{cc} & \theta^{\left(l+1\right)}\left(t\right)\\ & =\theta^{\left(l\right)}\left(t\right)-\kappa \nabla_{\theta }H\left(S^{\left(l\right)}\left(t\right),I^{\left(l\right)}\left(t\right),R^{\left(l\right)}\left(t\right),\theta^{\left(l\right)}\left(t\right),\lambda_{S}^{\left(l\right)}\left(t\right),\lambda_{I}^{\left(l\right)}\left(t\right),\lambda_{R}^{\left(l\right)}\left(t\right)\right). \end{array} \end{array}$$ (15)

Then, there exists a function *θ* such that the system (16) holds.

$$\begin{array}{c} \left\{ \begin{array}{cc} \beta^{\left(l+1\right)}\left(t\right) & =\beta^{\left(l\right)}\left(t\right)+\kappa \lambda_{S}^{\left(l\right)}\left(t\right)I^{\left(l\right)}\left(t\right)S^{\left(l\right)}\left(t\right)-\kappa \lambda_{I}^{\left(l\right)}\left(t\right)I^{\left(l\right)}\left(t\right)S^{\left(l\right)}\left(t\right),\\ c^{\left(l+1\right)}\left(t\right) & =c^{\left(l\right)}\left(t\right)+\kappa \frac{1}{\gamma }\lambda_{I}^{\left(l\right)}\left(t\right)I^{\left(l\right)}\left(t\right)-\kappa \frac{1}{\gamma }\lambda_{R}^{\left(l\right)}\left(t\right)I^{\left(l\right)}\left(t\right),\\ \theta (t)\in \Theta , & t\in (t_{h-1},t_{h}),h=1,\cdots ,H, \end{array}\right. \end{array}$$ (16)

The optimality system for the ARS model (8) consists of Eqs (2), (11) and (12). Drawing inspiration from the well-known Forward-Backward Method [28] for solving differential equations, we present a cost-effective defense algorithm, referred to as the CED algorithm and described in Algorithm 2, for solving the optimality system of the ARS model. In the algorithm, ∥ ⋅ ∥_2_ stands for the 2-norm of a vector.

**Algorithm 2 CED**

**Input:** An instance of the ARS model $M=(S_{0},I_{0},R_{0},T,\tilde{I},\bar{\beta },\bar{c},\gamma ,\iota )$. A desired state *ι*, *m* equivalent points within each interval (*t*_*h*−1_, *t*_*h*_), and a convergence error *ϵ*.

**Output:** The parameter *θ*.

1: *l* ← 0; *θ*^(0)^← all-zero control;

2: **repeat**

3:  *l*++;

4:  **for** *h* ← 1 to *H* **do**

5:   **for** *k* ← 0 to *m* − 1 **do**

6:    Forward calculate (*S*, *I*, *R*) according to the system (2) with (*S*^(*h*, *k* + 1)^, *I*^(*h*, *k* + 1)^, *R*^(*h*, *k* + 1)^) ← (*S*^(*h*, *k*)^, *I*^(*h*, *k*)^, *R*^(*h*, *k*)^);

7:   Update the initial condition with (*S*^(*h*, 0)^, *I*^(*h*, 0)^, *R*^(*h*, 0)^)←(*S*^(*h*−1,*m*)^, *I*^(*h*−1,*m*)^, *R*^(*h*−1,*m*)^);

8:  Set the initial data with $\left(\lambda_{S}^{\left(H,m\right)},\lambda_{I}^{\left(H,m\right)},\lambda_{R}^{\left(H,m\right)}\right)\leftarrow \left(0,\frac{\partial G(I\left(t_{H}\right))}{\partial I},0\right)$;

9:  **for** *h* ← 1 to *H* **do**

10:   **for** *k* ← *m* − 1 to 0 **do**

11:    Backward calculate (λ_*S*_, λ_*I*_, λ_*R*_) according to the first equation of system (11) with $\left(\lambda_{S}^{\left(h,k\right)},\lambda_{I}^{\left(h,k\right)},\lambda_{R}^{\left(h,k\right)}\right)\leftarrow \left(\lambda_{S}^{\left(h,k+1\right)},\lambda_{I}^{\left(h,k+1\right)},\lambda_{R}^{\left(h,k+1\right)}\right)$;

12:   Update the initial condition with $\left(\lambda_{S}^{\left(h,m\right)},\lambda_{I}^{\left(h,m\right)},\lambda_{R}^{\left(h,m\right)}\right)\leftarrow \left(0,\lambda^{\left(h+1,0\right)}+\frac{\partial L(I\left(t_{h}\right))}{\partial I},0\right)$;

13:  Calculate *θ* using the system (16) with *θ*^(*l*+1)^ ← *θ*^(*l*)^;

14: **until** ∥*θ*^(*l*)^ − *θ*^(*l*−1)^∥_2_ < *ϵ*;

15: return *θ*^(*l*)^.

### 4.2 Complexity analysis

#### Time complexity

When dealing with new test samples, we first obtain *k* reported infection states using the RIR algorithm and then calculate the distance of each test sample from all the training samples. Assuming there are *N* training samples and each sample has a dimension of *M*, the time complexity of executing the RIR algorithm is *O*(*kMN*). For each instance of an ARS model, we utilize the CED algorithm to determine the desired parameters. If an ARS model converges after *l* iterations with *W* equivalent points, the time complexity of running the CED algorithm is *O*(*lW*). Thus, the total time required is *O*(*kMN*+ *lW*).

#### Space complexity

The space complexity of the RIR algorithm primarily depends on the storage capacity required for the training samples. With *N* training samples, each having *M* features, the space complexity of running the RIR algorithm is *O*(*MN*). Regarding an instance of an ARS model, the space complexity of the CED algorithm is primarily determined by the storage capacity for the equivalent points. Since *W* equivalent points need storage, the space complexity of executing the CED algorithm is *O*(*W*). Therefore, the total space required is *O*(*MN*+ *W*).

## 5 Experiments and result analysis

In the previous section, we presented two algorithms (i.e., the RIR and CED algorithms) for solving the ARS model. This section is dedicated to examining the performance of the proposed algorithms on a desktop computer equipped with an Intel Core i5 2.5GHz CPU, 32GB DDR4 RAM, and 64-bit Windows 10 operating system.

First, dynamic programming provides an alternative approach to solving deterministic optimal control problems [29]. The dynamic programming solution to a class of continuous optimal control problems is divided into two steps. In the first step, the original problem is approximated by a discrete-type optimal control problem. In the second step, the discrete Hamilton-Jacobi-Bellman equation of the problem is solved recursively to obtain the discrete optimal control. In this paper, we compare dynamic programming using optimal static propagation parameters with the method described in this paper [30].

Second, we employ significance tests to determine whether the fitting errors for the propagation parameter and the expected infection state are statistically significant or merely a result of chance. Specifically, the following two hypotheses are established:

- Null Hypothesis (*H*_0_): The model’s fitting error is attributed to chance.
- Alternative Hypothesis (*H*_1_): The model’s fitting error is statistically significant.

We set the significance level *α* = 0.05. Following the CED algorithm, we randomly select ten groups $\tilde{I}$ in chronological order to obtain the fitted error samples. To decide between parametric and non-parametric tests, we subject the error samples to tests for normality and homogeneity of variance. Finally, the outcomes of the significance tests determine whether *H*_0_ is rejected or not.

### 5.1 Efficiency of the ARS model

To conduct our experiments, we simulated an IoT network evolutionary model to characterize the evolutionary process of expected states over time in the subsection. The reported state generated by the simulated model serves as the input for the CED algorithm, and the output of the CED algorithm is then compared with the simulated model parameters. The entire process is implemented in MATLAB R2021.

Similarly, let *S*(*t*), *I*(*t*), and R*(*t*) denote the proportion of IoT devices in the susceptible, infected, and recovered states at time *t*. The simulated IoT network evolutionary model is described as follows:

$$\begin{array}{c} \left\{ \begin{array}{cc} \frac{dS^{*}\left(t\right)}{dt} & =-\beta^{*}\left(t\right)I^{*}\left(t\right)S^{*}\left(t\right),\\ \frac{dI^{*}\left(t\right)}{dt} & =\beta^{*}\left(t\right)I^{*}\left(t\right)S^{*}\left(t\right)-\frac{1}{\gamma^{*}}c^{*}\left(t\right)I^{*}\left(t\right),\\ \frac{dR^{*}\left(t\right)}{dt} & =\frac{1}{\gamma^{*}}c^{*}\left(t\right)I^{*}\left(t\right),\\ S^{*}\left(0\right) & =S_{0}^{*},I^{*}\left(0\right)=I_{0}^{*},R^{*}\left(0\right)=R_{0}^{*}. \end{array}\right. \end{array}$$ (17)

**Experiment 1**. *Consider the following instance*.

$$\begin{array}{cc} M_{1} & =(0.9,0.1,0,100,\left(I^{*}\left(0\right),I^{*}\left(5\right),\cdots ,I^{*}\left(100\right)\right),\\ & 0.1,0.1,1,0.7I^{*}\left(100\right)), \end{array}$$

*where* $S_{0}^{*}=0.9$, $I_{0}^{*}=0.1$, $R_{0}^{*}=0$, β*(*t*) = 0.01(1 + *sin*(0.1*t*)), c*(*t*) = 0. *Additionally, let ϵ* = 10^−6^.

**Fig 3:**
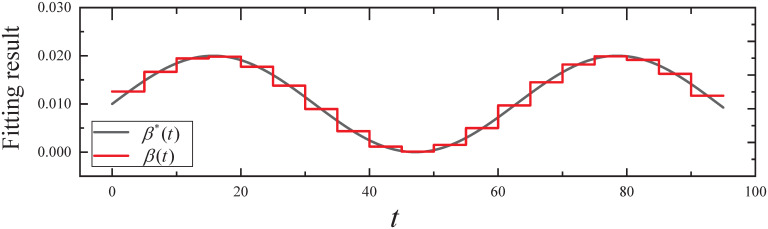
The fitting result of the propagation parameter in Experiment 1.

**Fig 4:**
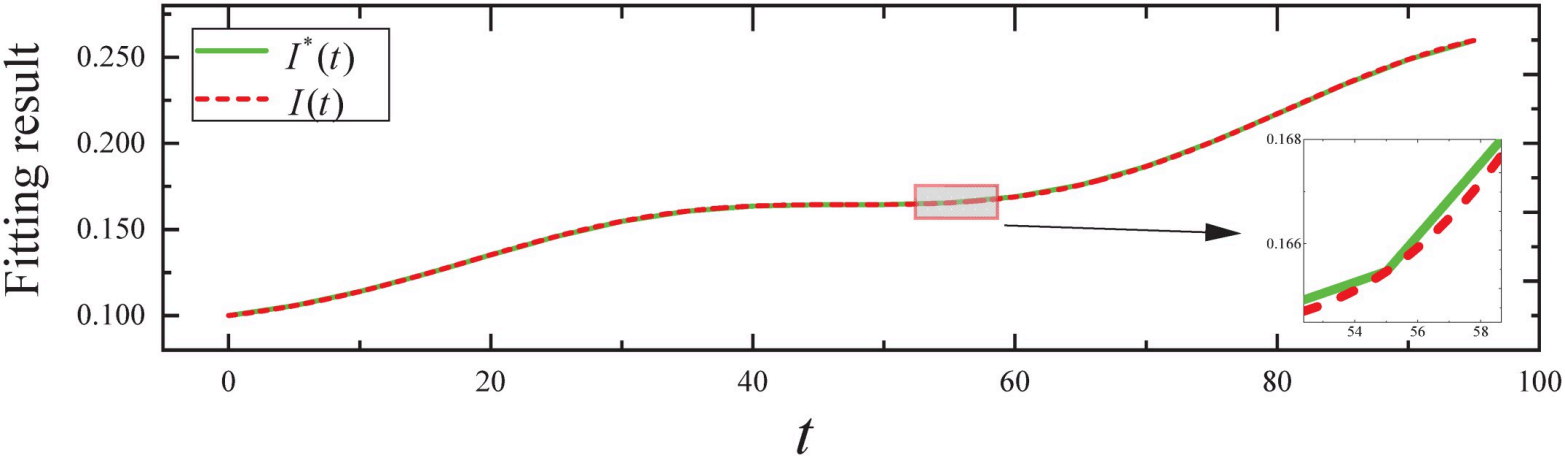
The fitting result of the expected infected state in Experiment 1.

By executing the CED algorithm on $\left(M_{1},\epsilon \right)$: (i) We obtain the fitting result of the propagation parameter *β*(*t*) for *t* ∈ [0, 95], which is plotted in Fig 3. From Fig 3, it can be seen that the propagation parameter *β*(*t*) fits the β*(*t*) of the simulated evolutionary model well. (ii) We obtain the fitting result of the expected infected state *I*(*t*) for *t* ∈ [0, 95], which is plotted in Fig 4. From Fig 4, it can be seen that infected state *I*(*t*) fits the I*(*t*) of the simulated evolutionary model well.

By executing the Wilcoxon signed-rank test, a nonparametric test, as neither the fitted error samples for the propagation parameters nor the fitted error samples for the expected states satisfy the normality and homogeneity of variance requirements: (i) The resulting *P*-value from the fitting errors of propagation parameters is 2.3202 × 10^−06^, which is less than *α*. Therefore, *H*_0_ is rejected, indicating that the fit of the propagation parameter is statistically significant. (ii) The resulting *P*-value from the fitting errors of expected infected states is 1.0345 × 10^−23^, which is less than *α*. Therefore, *H*_0_ is rejected, indicating that the fit of the expected infected state is statistically significant.

**Fig 5:**
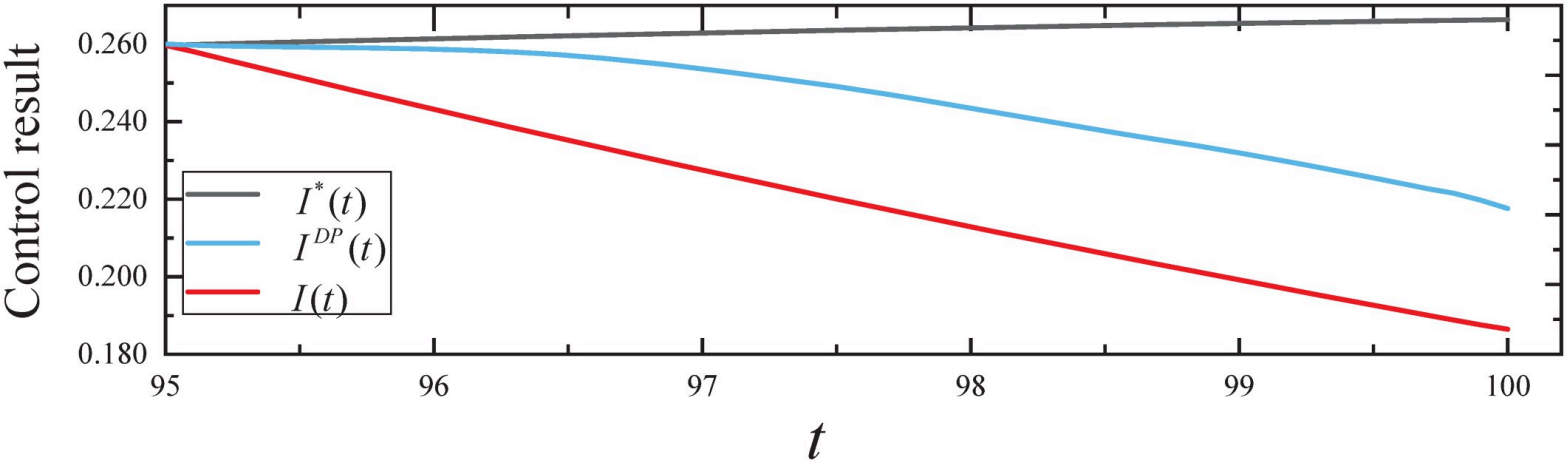
The control results in Experiment 1.

By executing the CED algorithm on $\left(M_{1},\epsilon \right)$: (i) We obtain the desired parameter *c*(*t*) for *t* ∈ [0, 100], the ARS *c*(*t*) is 0 for *t* ∈ [0, 95] and then increases to 0.066 for *t* ∈ (95, 100] to control botware propagation. (ii) We obtain the resulting controls *I*(*t*) from the CED algorithm and *I*^*DP*^(*t*) from dynamic programming, as well as no control I*(*t*) from the simulated evolutionary model, which are plotted in Fig 5. From Fig 5, it can be seen that the expected infected states *I*(*t*) and *I*^*DP*^(*t*) decrease with time *t*. In addition, I*(*T*) = 0.266, *I*^*DP*^(*T*) = 0.217 = 0.816I*(*T*), and *I*(*T*) = 0.186 = 0.699I*(*T*) indicate that the CED algorithm achieved a more desirable effect of control than the dynamic programming algorithm.

**Experiment 2**. *Consider the following instance*.

$$\begin{array}{cc} M_{2} & =(0.9,0.1,0,100,\left(I^{*}\left(0\right),I^{*}\left(5\right),\cdots ,I^{*}\left(100\right)\right),\\ & 0.1,0.1,1,0.7I^{*}\left(100\right)), \end{array}$$

*where* $S_{0}^{*}=0.9$, $I_{0}^{*}=0.1$, $R_{0}^{*}=0$, $\beta^{*}\left(t\right)=\frac{0.05e^{0.1t}}{1+e^{0.1t}}$, c*(*t*) = 0, *Additionally, let ϵ* = 10^−6^.

**Fig 6:**
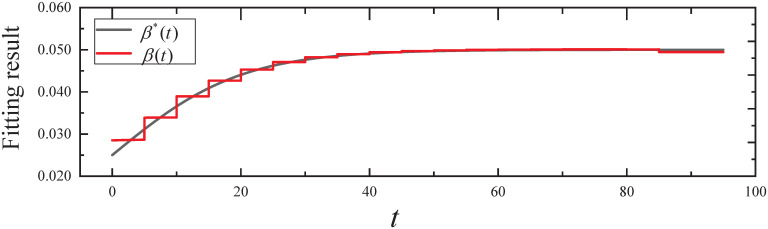
The fitting result of propagation parameter in Experiment 2.

**Fig 7:**
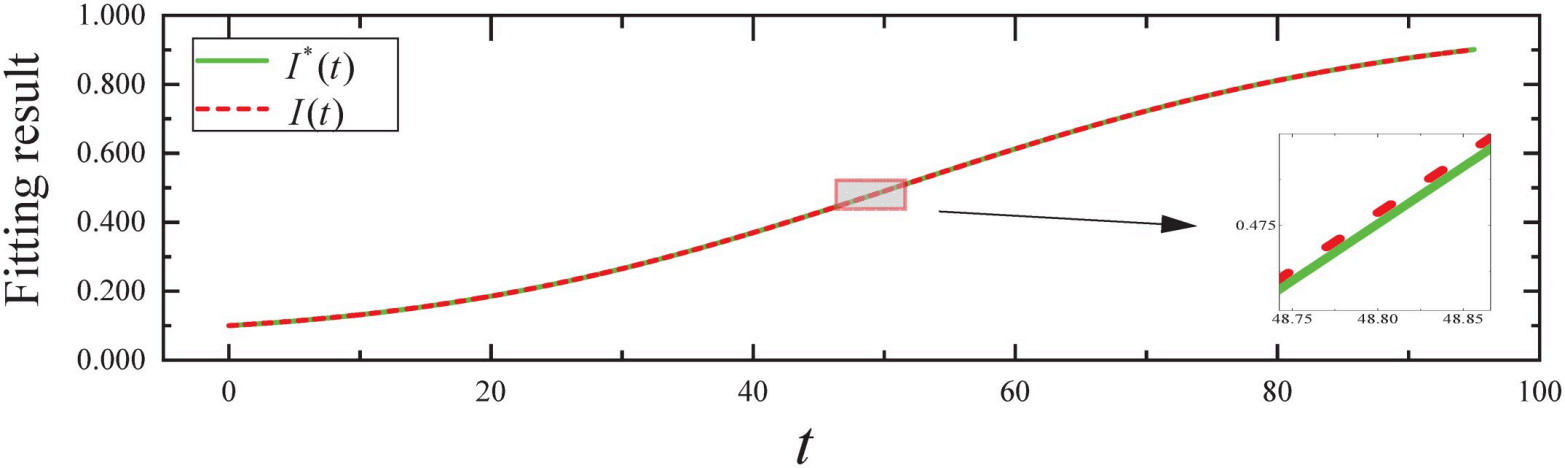
The fitting result of expected infected state in Experiment 2.

By executing the CED algorithm on $\left(M_{2},\epsilon \right)$: (i) We obtain the fitting result of the propagation parameter *β*(*t*) for *t* ∈ [0, 95], which is plotted in Fig 6. From Fig 6, it can be seen that the propagation parameter *β*(*t*) fits the β*(*t*) of the simulated evolutionary model well. (ii) We obtain the fitting result of the expected infected state *I*(*t*) for *t* ∈ [0, 95], which is plotted in Fig 7. From Fig 7, it can be seen that the infected state *I*(*t*) fits the I*(*t*) of the simulated evolutionary model well.

By executing the Wilcoxon signed-rank test, a nonparametric test, as neither the fitted error samples for the propagation parameters nor the fitted error samples for the expected states satisfy the normality and homogeneity of variance requirements: (i) The resulting *P*-value from the fitting errors of propagation parameters is 1.2013 × 10^−8^, which is less than *α*. Therefore, *H*_0_ is rejected, indicating that the fit of the propagation parameter is statistically significant. (ii) The resulting *P*-value from the fitting errors of expected infected states is 1.9684 × 10^−28^, which is less than *α*. Therefore, *H*_0_ is rejected, indicating that the fit of the expected infected state is statistically significant.

**Fig 8:**
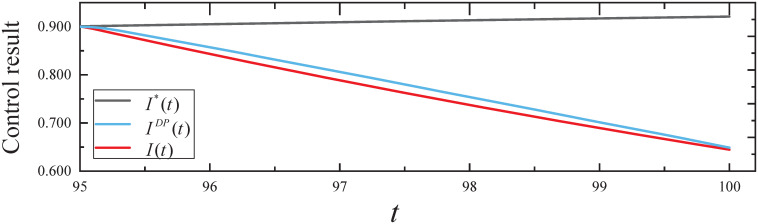
The control results in Experiment 2.

By executing the CED algorithm on $\left(M_{2},\epsilon \right)$: (i) We obtain the desired parameter *c*(*t*) for *t* ∈ [0, 100], the ARS *c*(*t*) is 0 for *t* ∈ [0, 95] and then increases to 0.067 for *t* ∈ (95, 100] to control botware propagation. (ii) We obtain the resulting controls *I*(*t*) from the CED algorithm and *I*^*DP*^(*t*) from dynamic programming, as well as no control I*(*t*) from simulated evolutionary model, which are plotted in Fig 8. From Fig 8, it can be seen that the expected infected states *I*(*t*) and *I*^*DP*^(*t*) decrease with time *t*. In addition, I*(*T*) = 0.921, *I*^*DP*^(*T*) = 0.648 = 0.704I*(*T*), and *I*(*T*) = 0.645 = 0.698I*(*T*) indicate that the CED algorithm achieved a more desirable effect of control than the dynamic programming algorithm.

In conclusion, the simulation experiments show that: (i) The CED algorithm can effectively fit and predict propagation. (ii) The CED algorithm exhibits good performance and achieves the goal by setting the parameters according to what has been learned.

### 5.2 Efficiency of the ARS model with case studies

To further demonstrate the efficiency of the ARS model, we analyze the ARS problem with two case studies in the subsection.

The first dataset used in Experiment 3 comes from MedBIoT [31], a medium-sized network that combines 83 real and emulated IoT devices. The dataset is split according to the traffic source (i.e., benign or malicious traffic), and it includes three prominent types of botnet malware: Mirai, BashLite, and Torii. We only use the original bulk pcap file of Mirai botnet malware traffic and benign traffic for feature extraction. We obtain the reported state $\tilde{I}=\left(0.012,0.048,0.12,0.169,0.205,0.289,0.289\right)$ according to our proposed algorithm (i.e., the RIR algorithm), which is implemented in Kali Linux using Python scripting.

**Experiment 3**. *Consider the following instance*.

$$\begin{array}{c} M_{3}=\left(0.988,0.012,0,6,\tilde{I},5,5,10,0.145\right). \end{array}$$

*Additionally, let ϵ* = 10^−6^.

**Fig 9:**
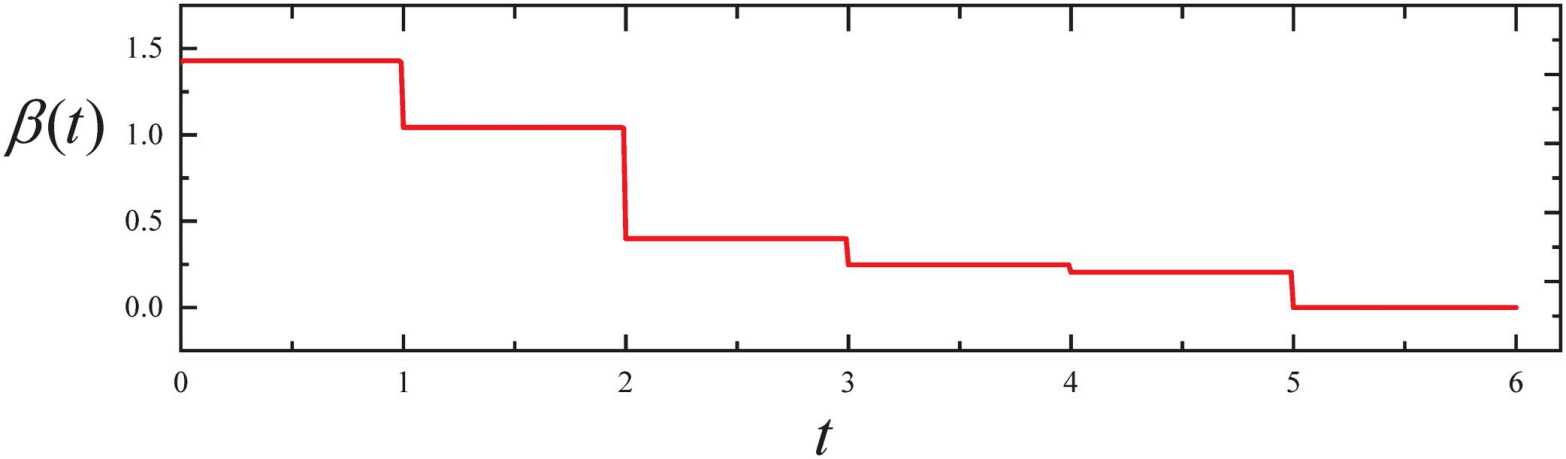
The resulting propagation parameter in Experiment 3.

By executing the CED algorithm on $\left(M_{3},\epsilon \right)$: (i) We obtain the propagation parameter *β*(*t*) for *t* ∈ [0, *T*], which is plotted in Fig 9. From Fig 9, it can be seen that *β*(*t*) decreases with the increase of *t*. (ii) We obtain the desired parameter *c*(*t*) for *t* ∈ [0, *T*]. The ARS *c*(*t*) is 0 for *t* ∈ [0, 5], and then increases to 3.051 for *t* ∈ (5, 6].

**Fig 10:**
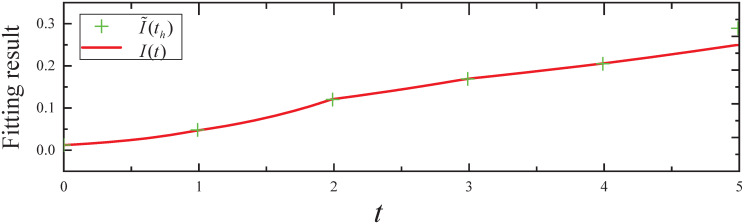
The fitting result of expected infected state in Experiment 3.

**Fig 11:**
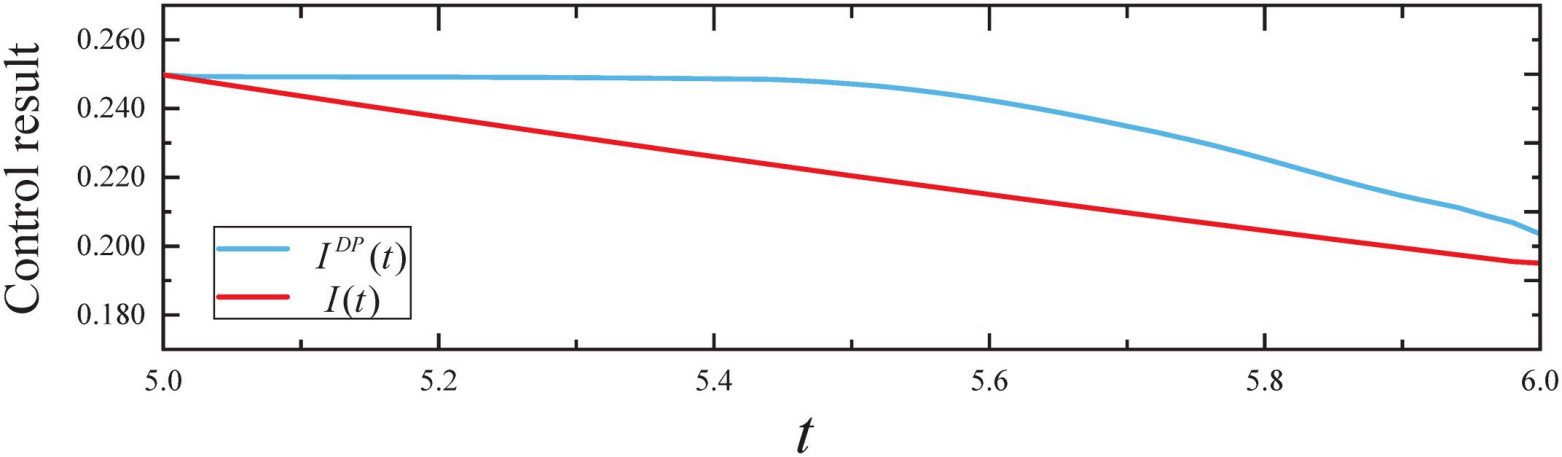
The control results in Experiment 3.

We obtain the expected infected state *I*(*t*) for *t* ∈ [0, 5], which is plotted in Fig 10. From Fig 10, it can be seen that: (i) The infected state *I*(*t*) fits the $\tilde{I}$ well for *t* ∈ [0, 5]. (ii) We obtain the resulting controls *I*(*t*) from the CED algorithm and *I*^*DP*^(*t*) from dynamic programming for *t* ∈ (5, 6], which are plotted in Fig 11. From Fig 11, it can be seen that the expected infected state *I*(*t*) and *I*^*DP*^(*t*) decrease with time *t*. In addition, *I*^*DP*^(*T*) = 0.204 = 0.71I*(*T*) and *I*(*T*) = 0.199 = 0.69I*(*T*) indicate that the CED algorithm achieved a more desirable effect of control than the dynamic programming algorithm.

Due to the small sample size in Experiment 3, we create a new set of bootstrap samples by pulling multiple samples from the original sample in a relaxed manner and then perform the Wilcoxon signed-rank test on each one. By executing the Wilcoxon signed-rank test, the resulting *P*-value from the fitting errors of expected infected states is 4.5831 × 10^−7^, which is less than *α*. Therefore, *H*_0_ is rejected, indicating that the fit of the expected infected state is statistically significant.

The second dataset used in Experiment 4 comes from TI-DNS [32], which contains ten days of real-world DNS traffic as well as nine different botnets. We analyze the traffic from the first day, where the reported state $\tilde{I}$ is calculated as the proportion of infected hosts over all hosts in each hour.

**Experiment 4**. *Consider the following instance*.

$$\begin{array}{c} M_{4}=\left(0.99,0.01,0,23,\tilde{I},5,5,10,\tilde{I}\left(t_{23}\right)\right). \end{array}$$

*Additionally, let ϵ* = 10^−6^.

**Fig 12:**
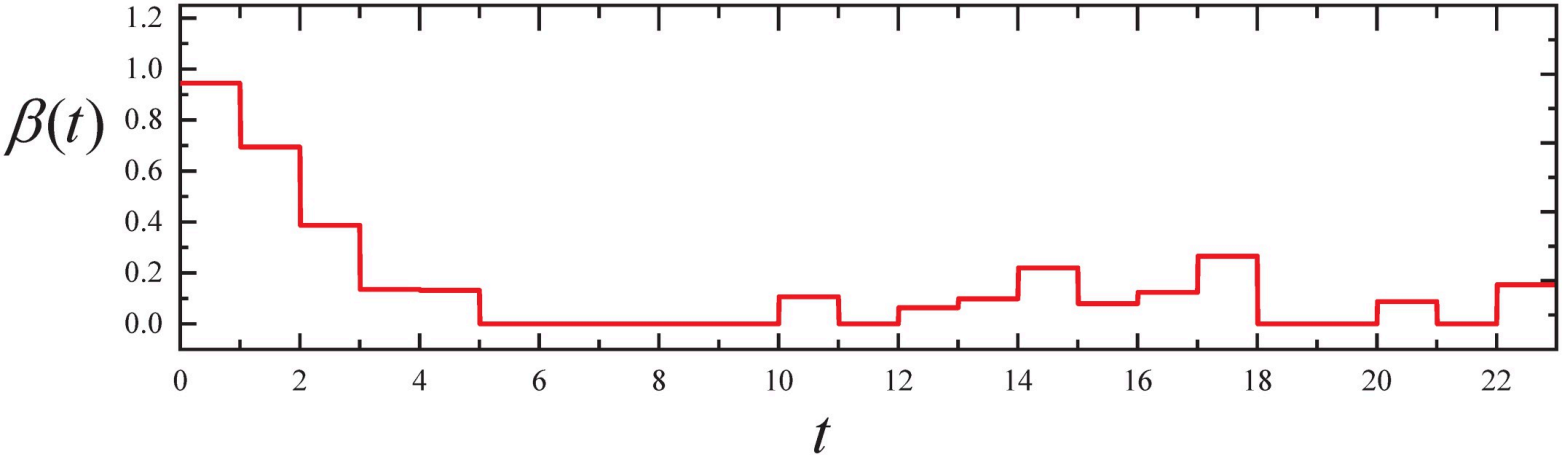
The resulting propagation parameter in Experiment 4.

**Fig 13:**
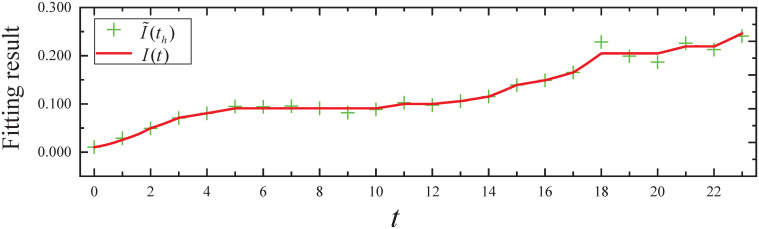
The fitting result of infection state in Experiment 4.

By executing the CED algorithm with no control on $\left(M_{4},\epsilon \right)$: (i) We obtain the propagation parameter *β*(*t*) for *t* ∈ [0, *T*], which is plotted in Fig 12. From Fig 12, it can be observed that the propagation rate shows a smaller increase from 12 p.m. to 6 p.m., and from 8 p.m. to 11 p.m., indicating that the users’ daily activities increase during these periods, allowing bots to connect as well. In the morning, from 0 a.m. to 3 a.m., the propagation rate shows a significant decrease, indicating that users start to rest during this period. (ii) We obtain the expected infected state *I*(*t*) for *t* ∈ [0, *T*], which is plotted in Fig 13. From Fig 13, it can be seen that the infected state *I*(*t*) fits the $\tilde{I}$ well for *t* ∈ [0, *T*].

By executing the Wilcoxon signed-rank test, the resulting *P*-value from the fitting errors of expected infected states is 2.7 × 10^−5^, which is less than *α*. Therefore, *H*_0_ is rejected, indicating that the fit of the expected infected state is statistically significant.

In conclusion, the case studies show that (i) the CED algorithm can effectively fit and predict propagation, and (ii) the CED algorithm has good performance, achieving the goal by setting the parameters according to what has been learned.

In addition, Table 2 gives the evaluation of Experiment 1 to Experiment 4, including execution time, memory usage, and iterations.

**Table 2:** The evaluation of experiments.

	Algorithm	Execution time	Memory usage	Iterations
Experiment 1	CED dynamic programming	29.634s 839.462s	9.629MB 847MB	4871 /
Experiment 2	CED dynamic programming	45.825s 726.29s	21.969MB 747MB	7443 /
Experiment 3	CED dynamic programming	35.862s 654.519s	2.797MB 675.1MB	9566 /
Experiment 4	CED dynamic programming	107.658s /	14.96MB /	8724 /

### 5.3 Influence of the DDoS stealthiness level

In this subsection, the experimental results are given to examine how the ARS *c*(*t*) is affected by the DDoS stealthiness level (i.e., *γ*) within the simulated model.

**Experiment 5**. *Let* Γ = {1, 1.1, ⋯, 2}. *Consider the following instance*.

$$\begin{array}{cc} M_{5} & =(0.9,0.1,0,100,\left(I^{*}\left(0\right),I^{*}\left(10\right),\cdots ,I^{*}\left(90\right)\right),\\ & 0.1,0.1,\gamma ,0.7I^{*}\left(100\right)),\;\gamma \in \Gamma \end{array}$$

*where* $S_{0}^{*}=0.9$, $I_{0}^{*}=0.1$, $R_{0}^{*}=0$, β*(*t*) = 0.01(1 + *sin*(0.1*t*)), c*(*t*) = 0. *Additionally, let ϵ* = 10^−6^.

**Fig 14:**
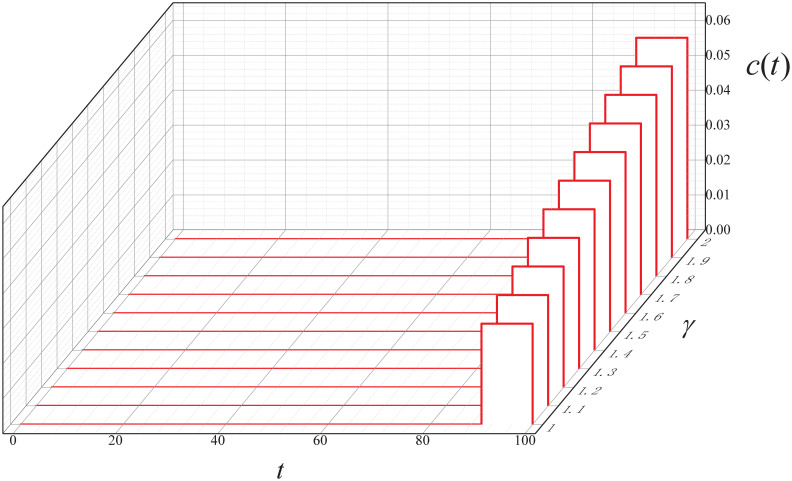
The control result in Experiment 5.

By executing the CED algorithm on $\left(M_{5},\epsilon \right)$, we attain the ARS *c*(*t*) versus the DDoS stealthiness level *γ*, which is plotted in Fig 14, it can be seen that the cost increases with the rise in the DDoS stealthiness level during the control period *t* ∈ (90, 100]. This finding suggests that IoT bots with higher stealthiness levels require greater costs for repair.

**Experiment 6**. *Let* Γ = {1, 1.1, ⋯, 2}. *Consider the following instance*.

$$\begin{array}{cc} M_{6} & =(0.9,0.1,0,100,\left(I^{*}\left(0\right),I^{*}\left(10\right),\cdots ,I^{*}\left(90\right)\right),\\ & 0.1,0.1,\gamma ,0.7I^{*}\left(100\right)),\;\gamma \in \Gamma \end{array}$$

*where* $S_{0}^{*}=0.9$, $I_{0}^{*}=0.1$, $R_{0}^{*}=0$, $\beta^{*}\left(t\right)=\frac{0.05e^{0.1t}}{1+e^{0.1t}}$, c*(*t*) = 0, *Additionally, let ϵ* = 10^−6^.

**Fig 15:**
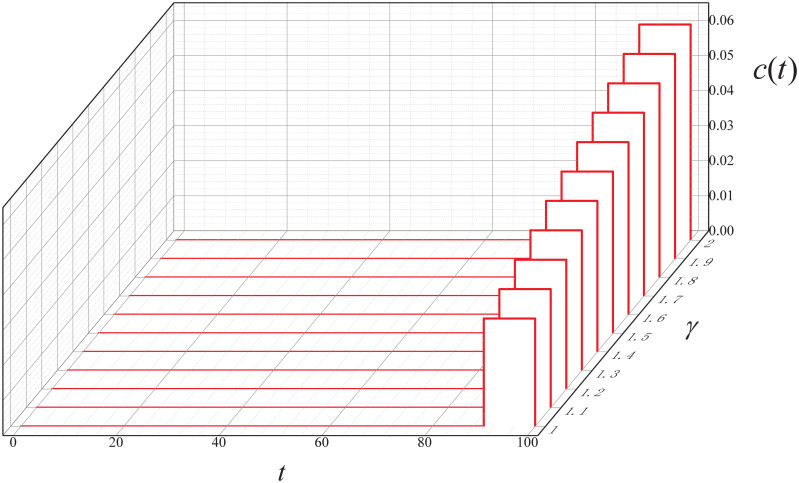
The control result in Experiment 6.

By executing the CED algorithm on $\left(M_{6},\epsilon \right)$, we attain the ARS *c*(*t*) versus the DDoS stealthiness level *γ*, which is plotted in Fig 15. It can be seen that the cost increases with the rise in the DDoS stealthiness level during control period *t* ∈ (90, 100]. This finding also suggests that IoT bots with higher stealthiness levels require greater costs for repair.

We conducted a total of 100 similar experiments. In each experiment, we observed that the cost increased with the rise in the DDoS stealthiness level during the control period. To reduce repair costs, companies should develop more efficient detection mechanisms.

## 6 Conclusions and prospect

In the context of defending against DDoS-capable IoT botnets from a repair perspective, a novel adaptive defense problem has been proposed. The problem has been converted into a data-driven optimal control problem that can learn and predict propagation parameters based on network traffic data. Algorithms have been presented to solve the control problem, and a cost-effective adaptive defense strategy has been obtained by executing the algorithms.

Several related problems are yet to be investigated. First, while a deterministic optimal control problem is presented in this paper, the reported state of the IoT network is not necessarily accurate and is determined by the performance of the detection model. Therefore, stochastic optimal control theory or other probabilistic optimization techniques can be considered in the future to solve the ARS problem [33]. Second, the IoT botware propagation model established in this paper is a simple *SIR* epidemic model. However, different epidemic models and more detailed propagation parameters involved in these models should be established according to different application scenarios [8, 34]. Moreover, data-driven optimal control theory may be adapted to tackle other problems, such as cyber virus containment [35, 36], information spread [37, 38], and cyber defense [39, 40]. In addition, due to the non-cooperative confrontation between attacker and defender, the IoT network evolutionary problem can be tackled from the perspective of non-cooperative game theory [41–43].
